# Publisher Correction: Programming nonreciprocity and reversibility in multistable mechanical metamaterials

**DOI:** 10.1038/s41467-021-25049-w

**Published:** 2021-07-29

**Authors:** Gabriele Librandi, Eleonora Tubaldi, Katia Bertoldi

**Affiliations:** 1 John A. Paulson School of Engineering and Applied Sciences, Harvard University, Cambridge, MA USA; 2 Department of Mechanical Engineering, University of Maryland, College Park, MD USA

**Keywords:** Mechanical engineering, Materials for devices, Applied physics

Correction to: *Nature Communications* 10.1038/s41467-021-23690-z, published online 8 June 2021.

The original version of this Article contained an error in Fig. 1c, in which two single-headed arrows were inadvertently illustrated as double-sided arrows. This has been corrected in both the PDF and HTML versions of the Article.

