# Supplementary material for: Programming nonreciprocity and reversibility in multistable mechanical metamaterials
Source: Nat Commun. 2021 Jun 8;12:3454. doi: 10.1038/s41467-021-23690-z (PMC8187725; doi:10.1038/s41467-021-23690-z)
Supplement: Supplementary file 1 — Supplementary Information [file 41467_2021_23690_MOESM1_ESM.pdf]

Supplementary Information for *Programming nonreciprocity and reversibility in multistable mechanical metamaterials*

Gabriele Librandi[1], Eleonora Tubaldi[2,*], and Katia Bertoldi [1,*]

[1]Harvard John A. Paulson School of Engineering and Applied Sciences,
Harvard University, Cambridge, MA 02138, USA
[2]Mechanical Engineering Department,
University of Maryland, College Park, MD 20742, USA
*To whom correspondence should be addressed;
E-mail: etubaldi@umd.edu, bertoldi@seas.harvard.edu

S1 Supplementary Discussion

S1.1 Fabrication

The structures considered in this study comprise a 1D array of shallow arches made out of metallic beams and connected via rotating hinges. All beams have width $b = 10$ mm, length $l \in [103.1, 105.0]$ mm and are made of spring steel shims (McMaster-Carr product ID: 9014K611) with thickness $h = 0.3048$ mm and Young's modulus $E = 170$ GPa, while all hinges are realized using Lego components. Specifically, as shown in Supplementary Fig. 1, the hinges comprise the following components: (i) a round brick 2×2 with axle hole (LEGO part 4249139); (ii) an axle (LEGO part 3705); (iii) axle and pin connectors angled at 180 degrees (LEGO part 32034). Note that in order to ensure a tight connection between the beam and the hinges, slits of 3 mm length and 0.33 mm width are cut into both arms using a vertical knee drilling milling machine with slitting saw (Vectrax); (iv) two bushes (LEGO part 32123); and (v) a round brick

4×4 with a center axle hole (LEGO part 4211097). Note that for a structure with N arches we use $N+1$ of these hinges with their 4×4 round brick connected and glued (using Krazy Glue *All purposes*) to a Lego plate (LEGO part 91405). The axels of the hinges (LEGO part 3705) are located at a distance $L = 120$ mm from each other.

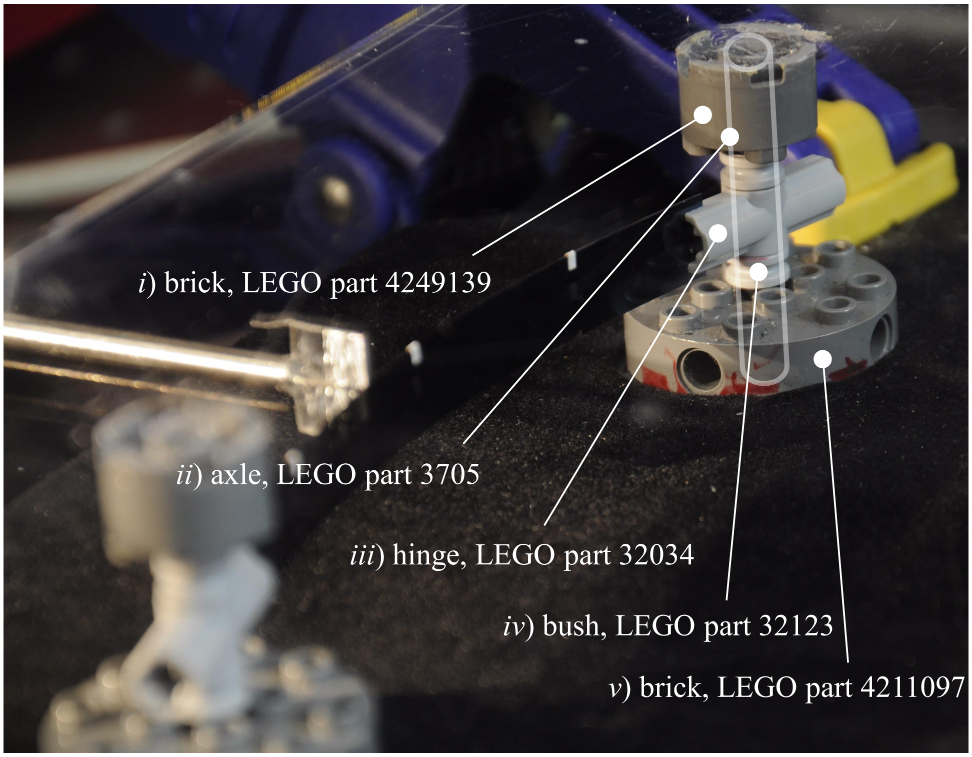

Supplementary Fig. 1: Hinges used in the experimental campaign. Picture of the hinge used to build our arches. The parts used to fabricate them are highlighted.

In the remaining part of this Section we first describe how we fabricate chains comprising only *elastically deformed* shallow arches and then report our manufacturing process to realize *plastically deformed* arches that are introduced into the chain in selected locations.

S1.2 Chains comprising only *elastically deformed* shallow arches

As shown in Supplementary Fig. 2, a chain comprising N elastically deformed arches is fabricated using the following 3 steps:

Step 1: N strips of length l (with $l \in [103.1, 105.0]$ mm) and width $b = 10$ mm are cut out of the steel shim by using a shearing tool. The strips are visually inspected to make sure they do not have any residual bending induced by the cutting procedure.

Step 2: Both ends of all steel strips are inserted into the cuts of the Lego connectors to form a 1D chain. Glue (Krazy Glue *All purposes*) is applied to prevent any sliding.

Step 3: An axial force is sequentially applied to all the strips to buckle them and form an array of N arches with end-to-end distance $L_{TOT} = N\,L$. Specifically, the axles (LEGO part 3705) are slid into the hinges connected to the arches (LEGO part 32034) and LEGO bushes (LEGO part 32123) are added on the axles to prevent other movements rather than rotation of the hinges. Lastly, an acrylic plate with LEGO round bricks (LEGO part 4249139) glued on it is fixed on the top to prevent bending of the axles. The array of arches is now ready to be tested.

S1.3 Chains comprising *elastically deformed* and *plastically deformed* shallow arches

As part of this study, we also fabricated structures comprising N_{el} elastically deformed arches and N_{pl} plastically deformed shallow arches introduced in selected locations. As shown in Supplementary Fig. 3, these structures are fabricated using the following 5 steps:

Step 1: N_{el} strips of length l =105.0 mm and width $b = 10$ mm are cut out of the steel shim by using a shearing tool. The strips are visually inspected to make sure they do not have any residual bending induced by the cutting procedure.

Step 2: N_{pl} strips of length l =105.0 mm and width $b = 10$ mm are cut with a shearing tool out of the steel shim. The strips are visually inspected to make sure they do not have any residual bending induced by the cutting procedure.

Step 3: Iron cylinders of diameter 34 and 25 mm are used to plastically deform the N_{pl} steel beams into a sinusoidal-like shape.

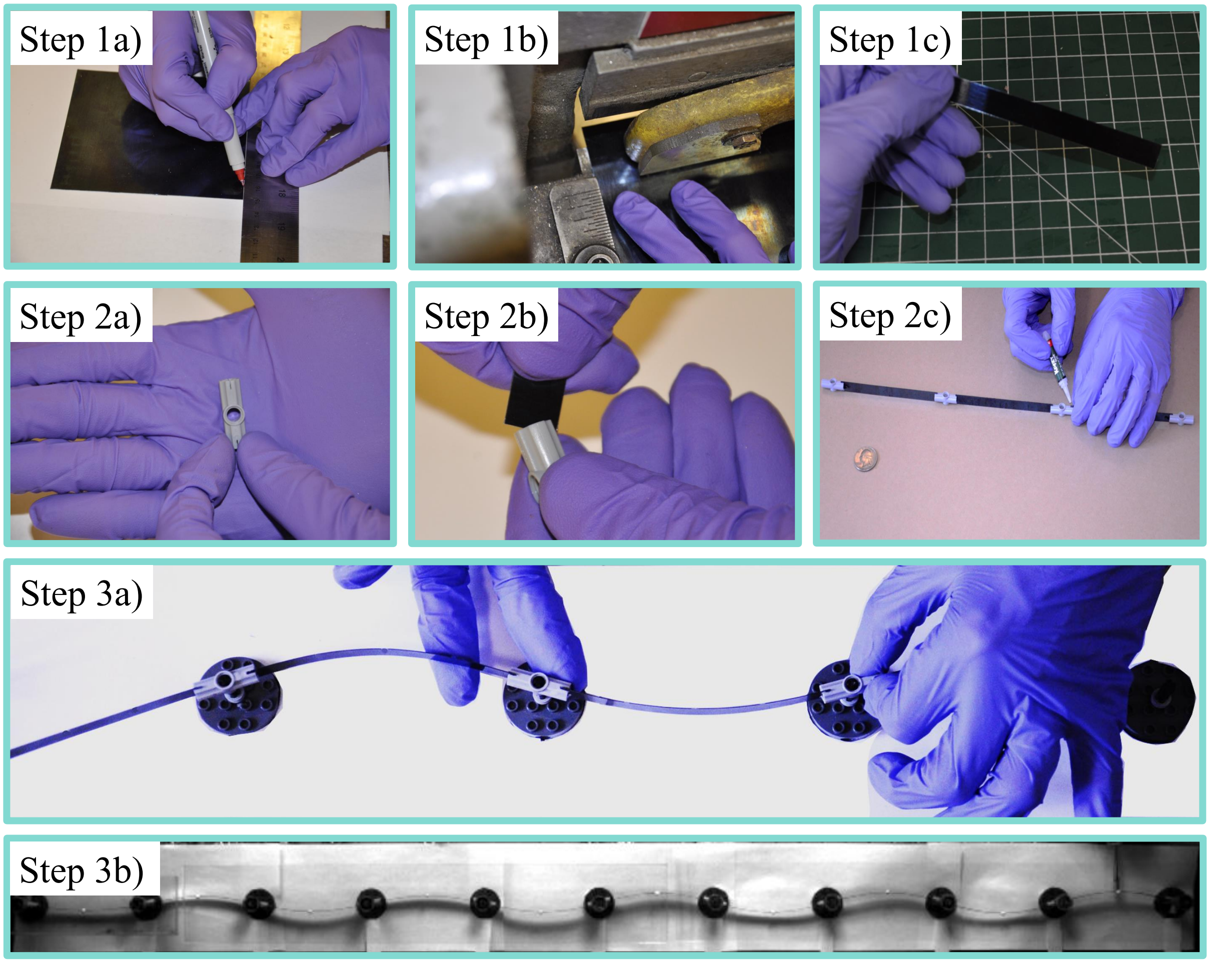

Supplementary Fig. 2: Fabrication. Fabrication steps to manufacture a chain comprising only *elastically deformed* shallow arches. **Step 1a-1c**, cutting the steel shims by using a shearing tool; **Step 2a-2c**, both ends of the steel strips are inserted into the Lego hinges and glue is applied to prevent sliding; **Step 3a-3b**, the array of arches is inserted into the Lego axles.

Step 4: The shape of the N_{pl} plastically deformed arch is visually compared with that of the target sinusoidal profile (which is laser cut out of an acrylic sheet with thickness 12.7 mm) to make sure the obtained shape is close enough to the desired one. In the unlikely event the arch has a very different shape with respect the benchmark, we either repeat Step 3 or start over from Step 2.

Step 5: Both ends of the N_{el} steel strips and the N_{pl} plastically deformed arches are inserted

into the cuts of the LEGO connectors to form a 1D chain (with the plastically deformed arches arranged in the desired location). Glue (Krazy Glue *All purposes*) is applied to prevent any sliding. The gluing is repeated on all the steel strips that are assembled together to form our array of arches.

Step 6: The N_{pl} plastically deformed arches are directly connected to the LEGO supports. An axial force is sequentially applied to the N_{el} strips to buckle them and form arches that are then connected to the Lego supports. Specifically, the axles (LEGO part 3705) are slid into the hinges connected to the arches (LEGO part 32034) and LEGO bushes (LEGO part 32123) are added on the axles to prevent other movements rather than rotation of the hinges. Lastly, an acrylic plate with LEGO round bricks (LEGO part 4249139) glued on it is fixed on the top to prevent bending of the axles. The array of arches is now ready to be tested.

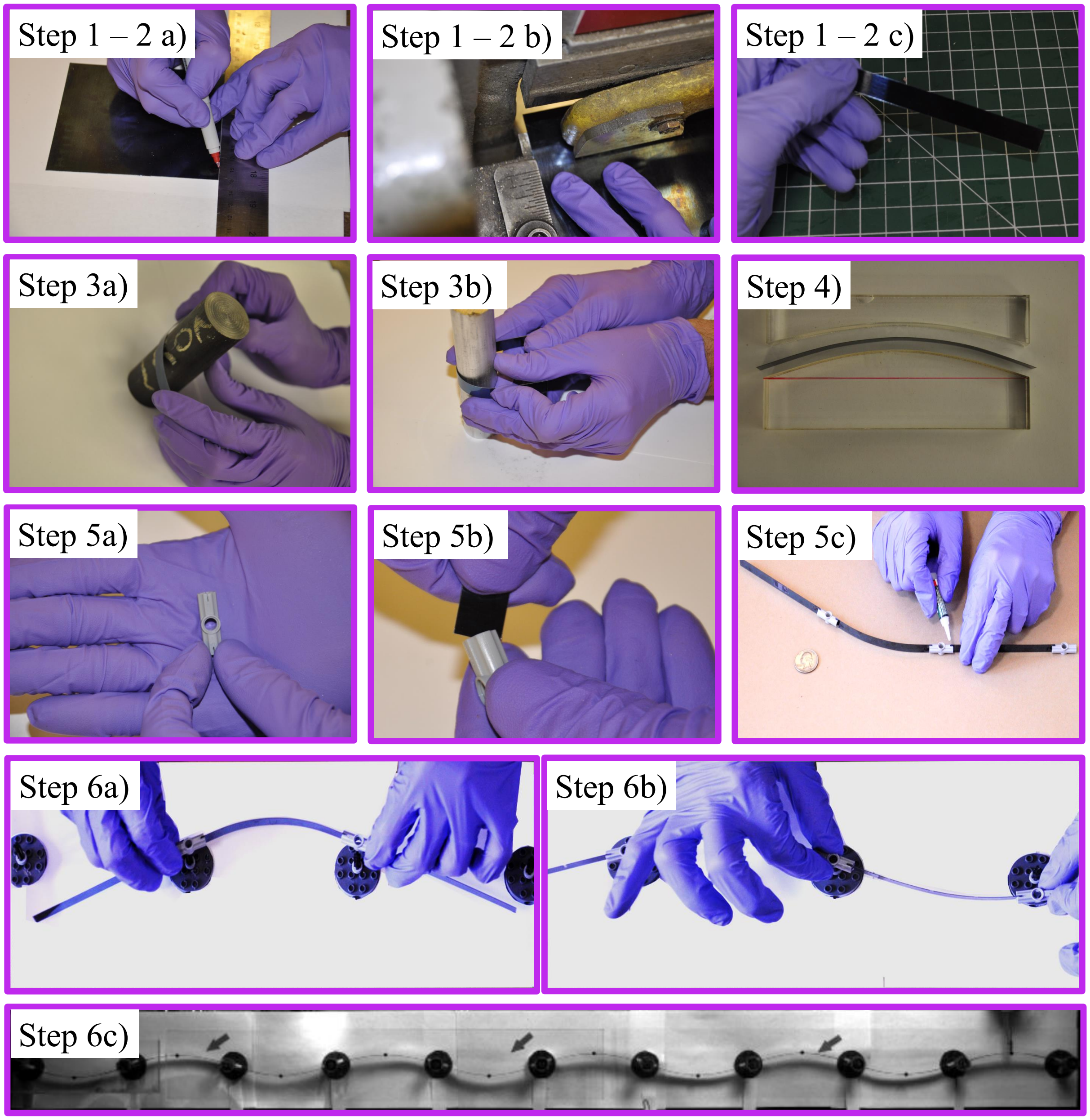

Supplementary Fig. 3: Fabrication. Fabrication steps to manufacture a chain comprising *elastically deformed* and *plastically deformed* shallow arches. **Step 1a-1c, Step 2a-2c**, cutting the steel shims by using a shearing tool; **Step 3a-3b**, iron cylinders are used to plastically deform the steel stripes; **Step 4**, the shape of the plastically deformed arch is visually compared with a target sinusoidal profile; **Step 5a-5c**, both ends of the steel strips and plastically deformed arches are inserted into the Lego hinges and glue is applied to prevent sliding; **Step 6a-6c**, the array of arches is inserted into the Lego axles.

S1.4 Testing

In all our tests we used an indenter (see inset in Supplementary Fig. 4) to push the central part of the first arch in the chain at a constant velocity of 15 mm/s (via a motorized translation stage - LTS300, Thorlabs). During our tests the reaction force is measured using a 10 lb load cell (LSB200 Miniature S-Beam Jr. Load cell, FUTEK Advanced Sensor Technology, Inc.). Moreover, a high speed camera (Photron Mini Series) is mounted above the testing area to track the displacements of the markers positioned at $L/2$ of each arch comprising our specimen. The camera records the entire experiment from the contact between the arch and the indenter up to the snapping of the entire array at a rate of 6400 fps.

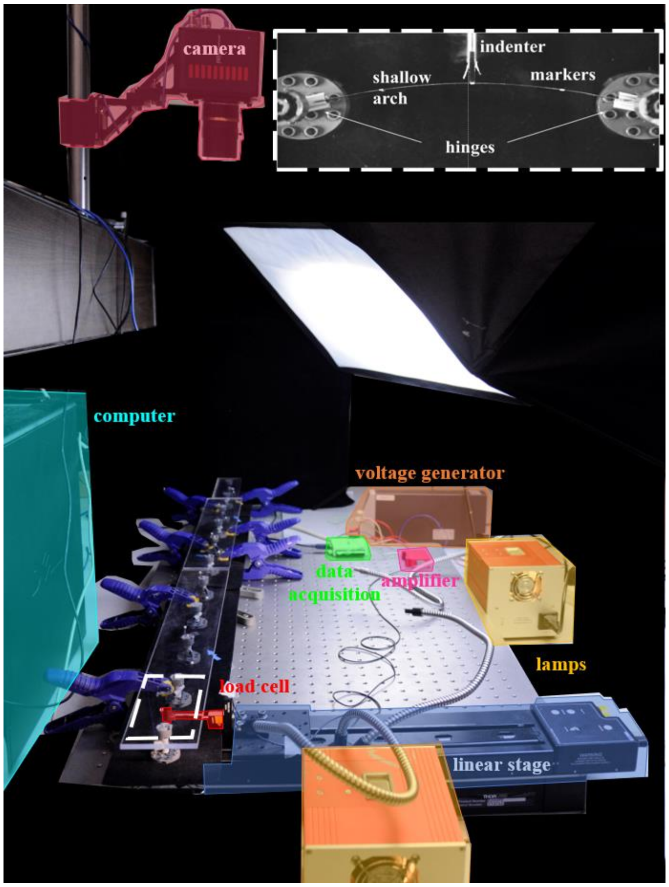

Supplementary Fig. 4: Experimental setup. Components of the experimental setup used to test our array of arches.

S1.5 Mathematical model

To get a better understanding of the dynamic response of our system, we establish a numerical model. As in our experiments, we consider a 1D array comprising N shallow arches connected via rotating hinges, see Supplementary Fig. 5. Focusing on the j-th arch ($j \in [1, N]$), we describe its initial shape as

$$w_j(x_j, t = 0) = e_j \sin\left(\frac{\pi x_j}{L}\right) \tag{S1}$$

where $x_j \in [0, L]$ (L denoting the span of the arches) and the rise e_j is positive if the arch is curved upwards and negative if the arch is curved downwards. We then apply a monotonically increasing displacement $d(t)$ ($d(t) < 0$ when pushing downwards) to the midpoint of the first arch in the array (note that the equations can be very easily adjusted to account for the loading of any other arch in the array) and solve for the time-dependent transverse profile of the j-th arch, $w_j(x_j,\, t)$ (which is defined positive for positive values of z). Towards this end, we use Euler-Bernoulli beam theory [1] to describe the behavior of the individual arches [2, 3, 4, 5, 6, 7, 8], so that their potential energy is given by

$$V_j = \frac{1}{2}EI\int_0^L \left(\frac{\partial^2 w_j}{\partial x_j^2} - \frac{d^2 w_{0j}}{dx_j^2}\right)^2 dx_j - \frac{1}{2}P_j\int_0^L \left(\frac{\partial w_j}{\partial x_j}\right)^2 dx_j + \frac{EA}{8L}\left[\int_0^L \left(\frac{\partial w_j}{\partial x_j}\right)^2 - \left(\frac{dw_{0j}}{dx_j}\right)^2 dx_j\right]^2 \tag{S2}$$

where w_{0_j} is the initial unstressed position of the midsurface of the j-th arch, P_j is the axial force applied to the j-th arch to elastically buckle it. Moreover, A and I are the area and moment of inertia of the cross section, E is the Young's modulus of the material.

Further, we impose continuity of rotations between neighboring elements [9, 10]

$$\left.\frac{\partial w_{j-1}}{\partial x_{j-1}}\right|_{x_{j-1}=L} = \left.\frac{\partial w_j}{\partial x_j}\right|_{x_j=0}. \tag{S3}$$

Importantly the constraints described by Eq. (S3) introduce concentrated moments, M_{Lj} and

M_{Rj}, at both ends of the j-th arch

$$M_{R_j} = M_{Rj}(t)\delta'(x_j - L)\,, \quad M_{L_j} = M_{Lj}(t)\delta'(x_j)\,, \tag{S4}$$

δ being the Dirac delta function, that satisfy

$$M_{R_{j-1}} = -M_{L_j}, \quad M_{R_j} = -M_{L_{j+1}}. \tag{S5}$$

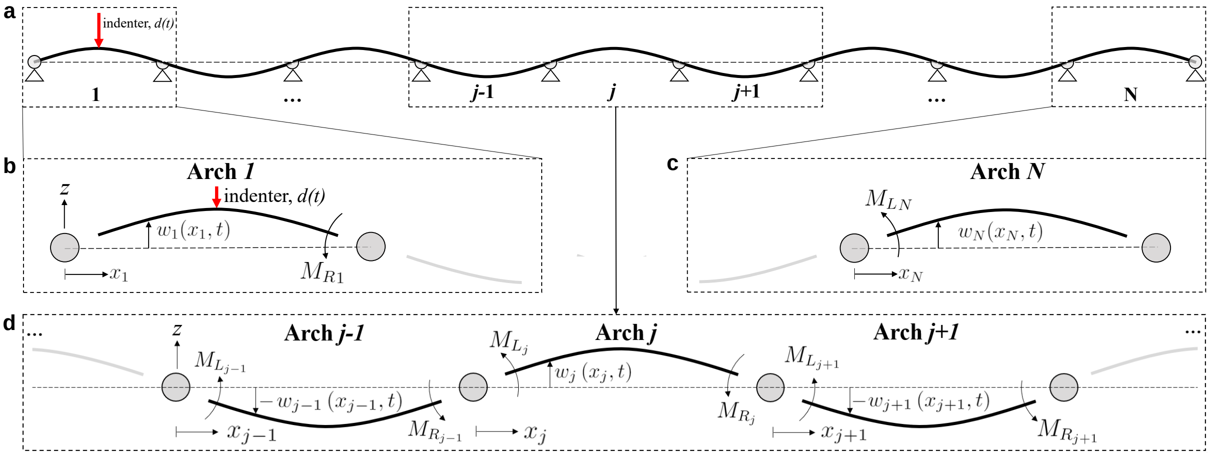

Supplementary Fig. 5: Schematic. **a**, Schematic of a 1D array comprising N shallow arches. **b**, Schematic of the arch where the indenter is applied. **c**, Schematic of the last arch in the chain. **d**, Schematic of a central portion of the chain where arches interact with neighboring elements both on the left and on the right hand side.

It follows that the the response of an array comprising N arches can be described as

$$\rho A \frac{\partial^2 w_j}{\partial t^2} + \beta \frac{\partial w_j}{\partial t} + EI\left(\frac{\partial^4 w_j}{\partial {x_j}^4} - \frac{d^4 w_{0_j}}{d{x_j}^4}\right) + p_j \frac{\partial^2 w_j}{\partial {x_j}^2} + Q_1 + M_{Rj} = 0 \qquad \text{for} \quad j = 1 \tag{S6a}$$

$$\rho A \frac{\partial^2 w_j}{\partial t^2} + \beta \frac{\partial w_j}{\partial t} + EI\left(\frac{\partial^4 w_j}{\partial {x_j}^4} - \frac{d^4 w_{0_j}}{d{x_j}^4}\right) + p_j \frac{\partial^2 w_j}{\partial {x_j}^2} + M_{Lj} + M_{Rj} = 0 \qquad \text{for} \quad j = 2, .., N-1 \tag{S6b}$$

$$\rho A \frac{\partial^2 w_j}{\partial t^2} + \beta \frac{\partial w_j}{\partial t} + EI\left(\frac{\partial^4 w_j}{\partial {x_j}^4} - \frac{d^4 w_{0_j}}{d{x_j}^4}\right) + p_j \frac{\partial^2 w_j}{\partial {x_j}^2} + M_{Lj} = 0 \qquad \text{for} \quad j = N \tag{S6c}$$

where $p_j = p_j(t)$ is the midplane force produced by the stretching of the middle surface of the j-th arch. Moreover, ρ and β are the volumetric density and the viscous damping coefficient, respectively. Further, Q_1 denotes the force measured at the midpoint of the first arch,

$$Q_1 = Q_1(t)\,\delta\left(x_1 - \frac{L}{2}\right) \tag{S7}$$

where δ is the Dirac delta function.

Additionally, since we are considering an indenter that controls the displacement of the first arch, we impose

$$w_1\left(\frac{L}{2}, t\right) = e_1 + d\,(t)\,, \tag{S8}$$

which holds true while the first arch is in contact with the indenter. Note that if the j-th arch is elastically deformed, the midplane force is given by [2, 7, 11]

$$p\,(t) = \left[\frac{EA}{L_{0j}}\left(L_{0j} - L\right) - \frac{EA}{2L}\int_0^L \left(\frac{\partial w_j}{\partial x_j}\right)^2 dx_j\right], \tag{S9}$$

and the initial unstressed position of the midsurface is

$$w_{0j} = 0, \tag{S10}$$

where L_{0j} denotes the length of the j-th beam in its undeformed configuration. Differently, if the j-th arch is plastically deformed, the midplane force is given by [2, 4, 12]

$$p\,(t) = -\frac{EA}{2L}\int_0^L \left[\left(\frac{\partial w_j}{\partial x_j}\right)^2 - \left(\frac{dw_{0j}}{dx_j}\right)^2\right] dx_j, \tag{S11}$$

and the initial unstressed position of the midsurface is

$$w_{0j}\,(x_j) = e_j \sin\left(\frac{\pi x_j}{L}\right). \tag{S12}$$

Importantly, for our system the deformed shape $w_j(x_j, t)$ can be expressed as a series of sine functions [2]

$$w_j(x_j,t) = w_{0j}(x_j) + \sum_{n=1}^{N_t} \psi_{nj}(t) \sin\left(\frac{n\pi x_j}{L}\right). \tag{S13}$$

If the j-th arch is an elastically deformed one, substitution Eqs. (S9), (S10), and (S13) into Eqs. (S6), multiplication of all terms by $\sin(m\pi x_j/L)$ (m being an integer, $m = 1, .., N_t$) and integration with respect to x_j from 0 to L yields

$$\frac{\rho A}{2}\ddot{\psi}_{nj} + \frac{\beta}{2}\dot{\psi}_{nj} + \frac{EI}{2}\left(\frac{n\pi}{L}\right)^4 \psi_{nj} - \frac{EAL}{2}\left(\frac{n\pi}{L}\right)^2 \left(\frac{L_{0j}-L}{L} - \frac{1}{4}\sum_{k=1}^{N_t}\left(\frac{k\pi}{L}\right)^2 \psi_{kj}^2\right)\psi_{nj} + q_n + m_{R_{nj}} = 0$$
$$\text{for} \quad j = 1, \quad n = 1, ..., N_t \tag{S14a}$$

$$\frac{\rho A}{2}\ddot{\psi}_{nj} + \frac{\beta}{2}\dot{\psi}_{nj} + \frac{EI}{2}\left(\frac{n\pi}{L}\right)^4 \psi_{nj} - \frac{EAL}{2}\left(\frac{n\pi}{L}\right)^2 \left(\frac{L_{0j}-L}{L} - \frac{1}{4}\sum_{k=1}^{N_t}\left(\frac{k\pi}{L}\right)^2 \psi_{kj}^2\right)\psi_{nj} + m_{R_{nj}} + m_{L_{nj}} = 0$$
$$\text{for } j \in [2, ..., N-1], \quad n = 1, ..., N_t \tag{S14b}$$

$$\frac{\rho A}{2}\ddot{\psi}_{nj} + \frac{\beta}{2}\dot{\psi}_{nj} + \frac{EI}{2}\left(\frac{n\pi}{L}\right)^4 \psi_{nj} - \frac{EAL}{2}\left(\frac{n\pi}{L}\right)^2 \left(\frac{L_{0j}-L}{L} - \frac{1}{4}\sum_{k=1}^{N_t}\left(\frac{k\pi}{L}\right)^2 \psi_{kj}^2\right)\psi_{nj} + m_{L_{nj}} = 0$$
$$\text{for} \quad j = N, \quad n = 1, ..., N_t \tag{S14c}$$

where

$$q_n = Q\sin\left(\frac{n\pi}{2}\right), \quad m_{R_{nj}} = -M_{Rj}\left(\frac{n\pi}{L}\right)\cos(n\pi), \quad m_{L_{nj}} = -M_{Lj}\left(\frac{n\pi}{L}\right). \tag{S15}$$

Moreover, by substituting Eqs. (S10) and (S13) into Eq. (S8) we obtain

$$-d(t) - e_1 + \sum_{n=1}^{\|(N_t+1)/2\|} -(-1)^n \psi_{2n-1} = 0. \tag{S16}$$

Differently, if the j-th arch is a plastically deformed one, substitution of Eqs. (S11), (S12), and (S13) into Eqs. (S6b)-(S6c), multiplication of all terms by $\sin(m\pi x_j/L)$ (m being an integer, $m = 1, .., N_t$) and integration with respect to x_j from 0 to L yields

$$\frac{\rho A}{2}\ddot{\psi}_{nj} + \frac{\beta}{2}\dot{\psi}_{nj} + \frac{EI}{2}\left(\frac{\pi}{L}\right)^4\psi_{nj} + \frac{EA}{4L}\left(\frac{\pi}{L}\right)^2\left(\frac{2e_j\pi}{L}\psi_{nj} + \sum_{k=1}^{N_t}\left(\frac{k\pi}{L}\right)^2\psi_{kj}^2\right)(\psi_{nj}+e_j) + m_{R_{nj}} + m_{L_{nj}} = 0$$

$$\text{for } j \in [2,...,N-1], \quad n=1$$

$$\frac{\rho A}{2}\ddot{\psi}_{nj} + \frac{\beta}{2}\dot{\psi}_{nj} + \frac{EI}{2}\left(\frac{n\pi}{L}\right)^4\psi_{nj} + \frac{EA}{4L}\left(\frac{n\pi}{L}\right)^2\left(\frac{2e_j\pi}{L}\psi_{nj} + \sum_{k=1}^{N_t}\left(\frac{k\pi}{L}\right)^2\psi_{kj}^2\right)\psi_{nj} + m_{R_{nj}} + m_{L_{nj}} = 0$$

$$\text{for } j \in [2,...,N-1], \quad n=2,...,N_t \tag{S17a}$$

$$\frac{\rho A}{2}\ddot{\psi}_{nj} + \frac{\beta}{2}\dot{\psi}_{nj} + \frac{EI}{2}\left(\frac{\pi}{L}\right)^4\psi_{nj} + \frac{EA}{4L}\left(\frac{\pi}{L}\right)^2\left(\frac{2e_j\pi}{L}\psi_{nj} + \sum_{k=1}^{N_t}\left(\frac{k\pi}{L}\right)^2\psi_{kj}^2\right)(\psi_{nj}+e_j) + m_{L_{nj}} = 0$$

$$\text{for } \quad j=N, \quad n=1$$

$$\frac{\rho A}{2}\ddot{\psi}_{nj} + \frac{\beta}{2}\dot{\psi}_{nj} + \frac{EI}{2}\left(\frac{n\pi}{L}\right)^4\psi_{nj} + \frac{EA}{4L}\left(\frac{n\pi}{L}\right)^2\left(\frac{2e_j\pi}{L}\psi_{nj} + \sum_{k=1}^{N_t}\left(\frac{k\pi}{L}\right)^2\psi_{kj}^2\right)\psi_{nj} + m_{L_{nj}} = 0$$

$$\text{for } \quad j=N, \quad n=2,...,N_t \tag{S17b}$$

where $m_{R_{nj}}$, and $m_{L_{nj}}$ are given by Eq. (S15). Note that, since in all our analyses the first arch in the chain is always an elastically deformed one, in Eqs. (S17) we don't not include the case $j = 1$.

Eqs. (S3), (S5), (S14), (S16), and (S17) are solved by numerical integration using the Runge-Kutta method (via the ODE45 function in Matlab) to obtain $\psi_{nj}(t)$. Note that in all our analyses we use $\rho = 7850\ kg/m^3$, $A = 3.05\ mm^2$, $E = 170\ GPa$, $I = 0.02\ mm^4$ (all values that are measured), $\beta = 6.71 \cdot 10^{-1}\ kg/(m \cdot s)$ for chains comprising only elastically deformed shallow arches and $\beta = 1.41\ kg/(m \cdot s)$ for chains comprising both elastically and plastically deformed shallow arches (note that these values are chosen to better capture the response observed in our tests).

At this point it is important to emphasize that Eqs. (S8) and (S14a) describe the behavior of the first arch when this is in contact with the indenter ($i.e.$ until $|Q_1(t)| > 0$) [2]. When $|Q_1(t)| = 0$ the first arch leaves the indenter, Eq. (S8) does not hold true anymore and Eq. (S14a) simplify to

$$\frac{\rho A}{2}\ddot{\psi}_{nj} + \frac{\beta}{2}\dot{\psi}_{nj} + \frac{EI}{2}\left(\frac{n\pi}{L}\right)^4 \psi_{nj} - \frac{EAL}{2}\left(\frac{n\pi}{L}\right)^2 \left(\frac{L_{0j}-L}{L} - \frac{1}{4}\sum_{k=1}^{N_t}\left(\frac{k\pi}{L}\right)^2 \psi_{k_j}^{\;2}\right)\psi_{nj} + m_{R_{nj}} = 0$$
$$\text{for} \quad j = 1, \quad n = 1, ..., N_t \tag{S18a}$$

The system comprising Eqs. (S3),(S5), (S18), Eqs. (S14b)-(S14c) for elastically deformed arches and Eqs. (S17) for plastically deformed arches is again numerically integrated using the Runge-Kutta method (via the ODE45 function in Matlab) with initial conditions (positions and velocities) given by Eqs. (S16) and Eqs. (S14) or Eqs. (S17) for elastically and plastically deformed arches, respectively.

To determine the number N_t of modes required for our model to accurately capture the response of our chains, we first focus on an array comprising $N = 3$ elastically deformed shallow arches with $e_j = 12.4$ mm. When considering $N_t = 5$ modes, we find that both ψ_4 and ψ_5 are negligible with respect the first three modes (i.e. ψ_j with $j = 1, 2, 3$) during the entire simulation (see Supplementary Fig. 6). As such, these results indicate that $N_t = 3$ modes are sufficient to capture the response of chains comprising elastically deformed arches. Next, we focus on an array comprising 3 elastically deformed arches and a plastically deformed one. As shown in 7, also for this case ψ_4 and ψ_5 are negligible during the entire simulation. Therefore, $N_t = 3$ modes are sufficient to capture also the response of chains comprising both elastically and plastically deformed arches.

Finally, we note that substitution of Eq. (S13) into Eq. (S2) yields

$$V_j = \frac{\pi^4 EI}{4L^3}\sum_{n=1}^{N_t} n^4\psi_{nj}^2 - EA\frac{\pi^2}{4L}\frac{L_0 - L}{L_0}\sum_{n=1}^{N_t} n^2\psi_{nj}^2 + \frac{\pi^4 EA}{32L^3}\left[\sum_{n=1}^{N_t} n^2\psi_{nj}^2\right]^2 \tag{S19}$$

for elastically deformed arches and

$$V_j = \frac{\pi^4 EI}{4L^3}\sum_{n=1}^{N_t} n^4\psi_{nj}^2 + \frac{\pi^4 EA}{32L^3}\left[2w_{0j}\psi_{1j} + \sum_{n=1}^{N_t} n^2\psi_{nj}^2\right]^2 \tag{S20}$$

for plastically deformed ones. Eqs. (S19) and (S20) with $N_t = 3$ are used to calculate the energy landscapes for the elastically and plastically deformed arches shown in Figs. 2f and 4a of the main text.

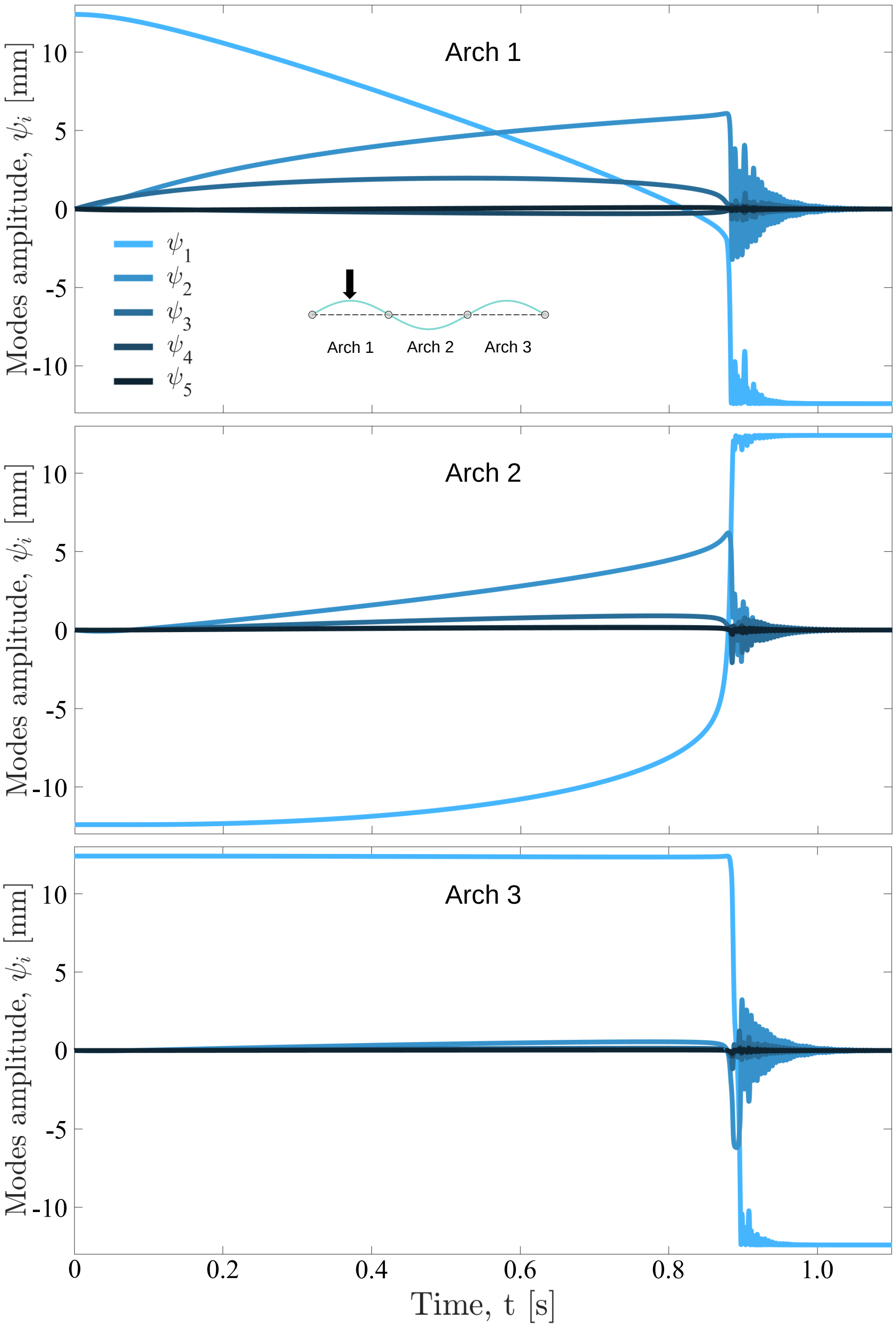

Supplementary Fig. 6: Effect of N_t on the response of an array comprising only elastic arches. Convergence analysis for a 1D array with $N = 3$ elastically deformed shallow arches. For all the three arches both the fourth, ψ_4, and the fifth, ψ_5, modes are negligible.

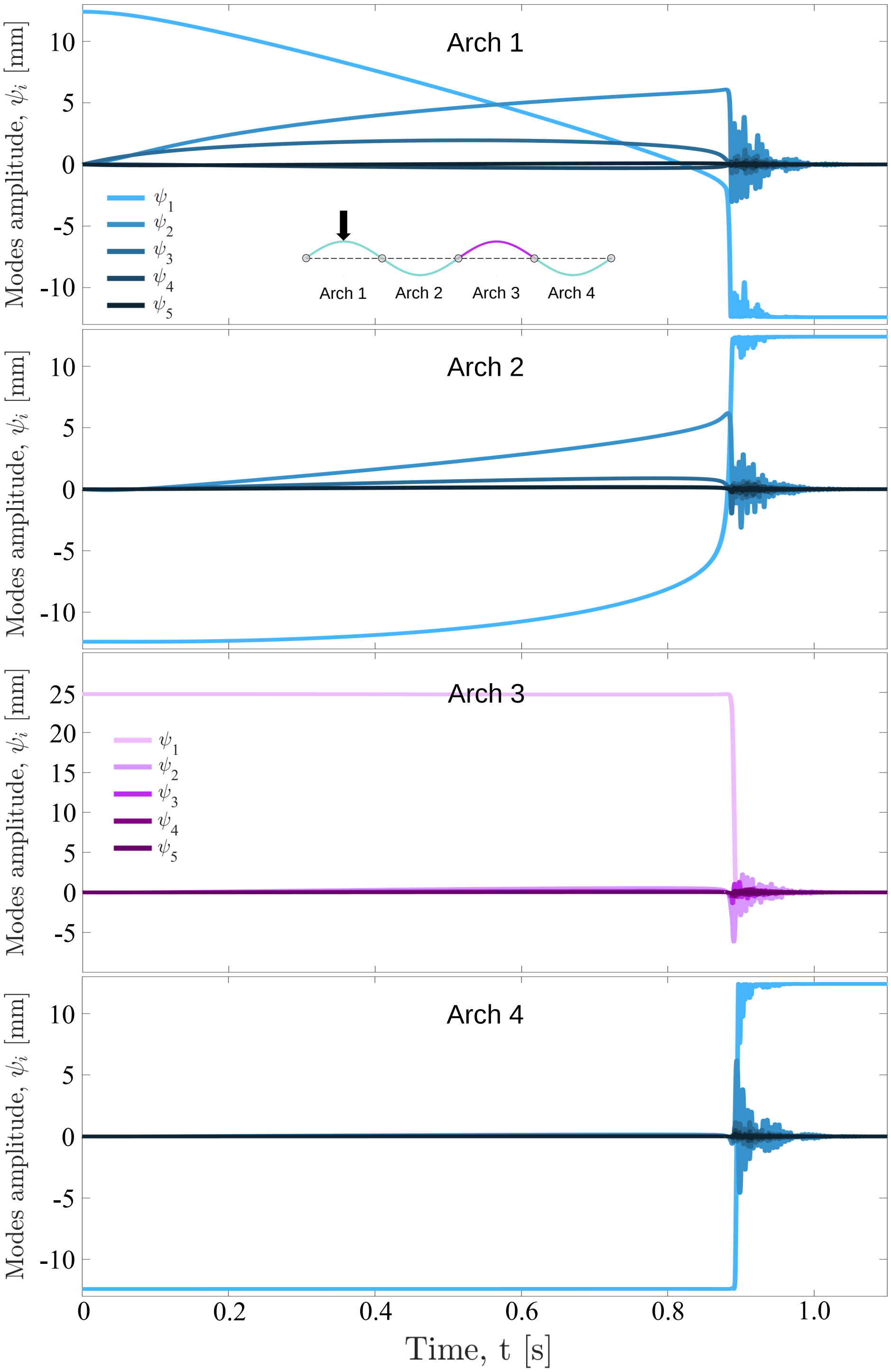

Supplementary Fig. 7: Effect of N_t on the response of an array comprising elastic and plastic arches. Convergence analysis for a 1D array with 3 elastically deformed shallow arches and one plastically deformed one. For all the three arches both the fourth, ψ_4, and the fifth, ψ_5, modes are negligible.

S1.6 Additional results

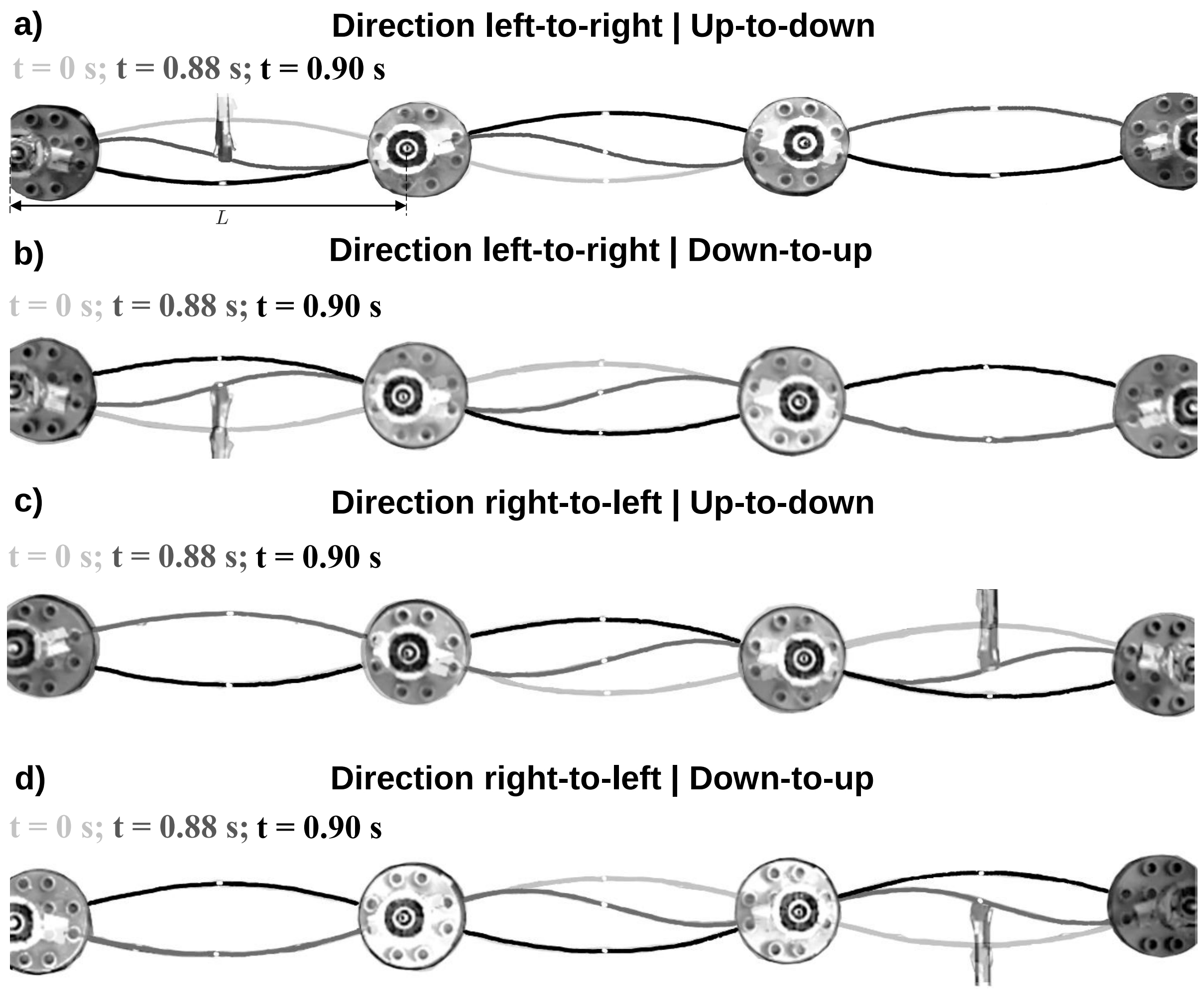

Supplementary Fig. 8: Symmetric elements – Symmetric array. Chain comprising three identical arches with rise $e_j = 12.4$ mm (j = 1,2,3) and symmetric energy wells. **a**, Signal propagation left-to-right by exciting the leftmost arch from up-to-down. **b**, Signal propagation left-to-right by exciting the leftmost arch from down-to-up. **c**, Signal propagation right-to-left by exciting the rightmost arch from up-to-down.**d**, Signal propagation right-to-left by exciting the rightmost arch from down-to-up.

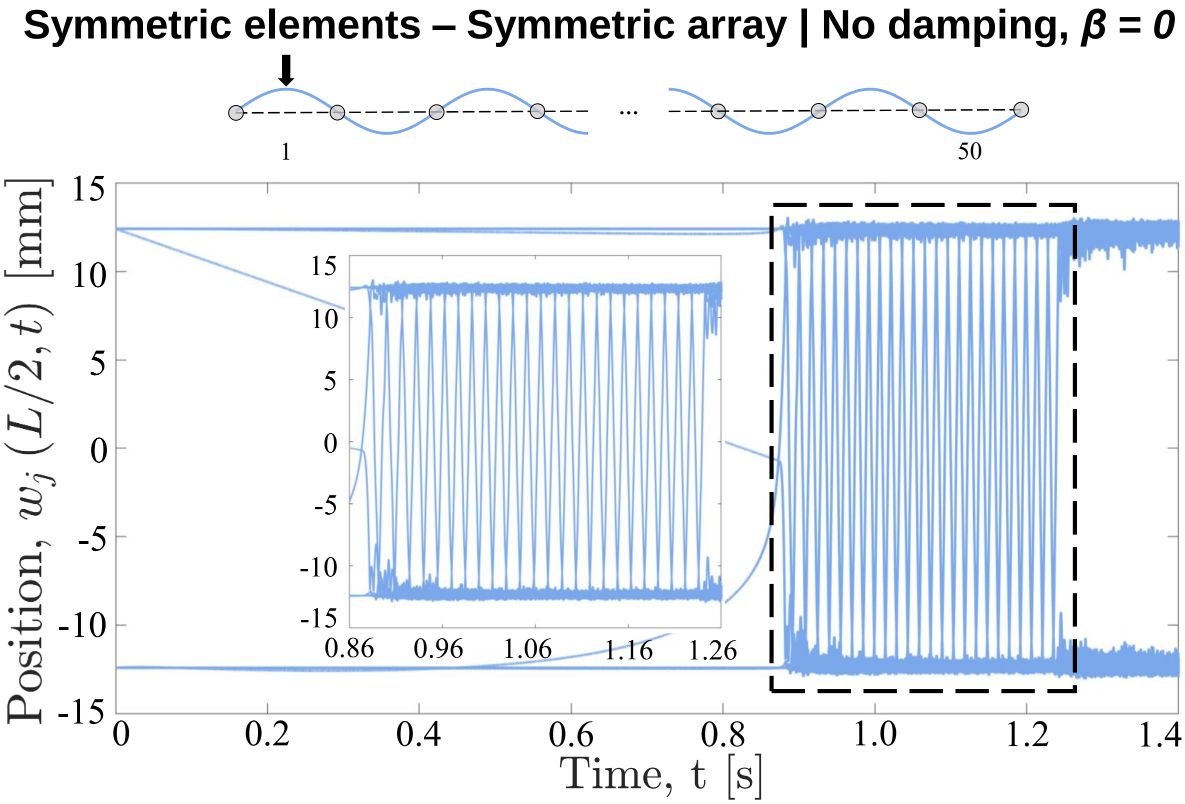

Supplementary Fig. 9: Symmetric elements – Symmetric array: response of an ideal chain with no damping. Numerical simulation of an array comprising $N = 50$ elastically deformed shallow arches all with rise $e_j = 12.4$ mm in the absence of damping (i.e. $\beta = 0$). An ideal system with all identical elastic elements and no damping can potentially sustain a transition wave over arbitrary distances.

	$w_j(L/2, 0)$ [mm]
Arch 1	12.13
Arch 2	11.64
Arch 3	11.00
Arch 4	10.63
Arch 5	9.88
Arch 6	9.59
Arch 7	9.03
Arch 8	8.49
Arch 9	7.92
Arch 10	7.51

Supplementary Table 1: Measured rises of the elastically deformed shallow arches in the chain considered in Fig. 3 of the main text.

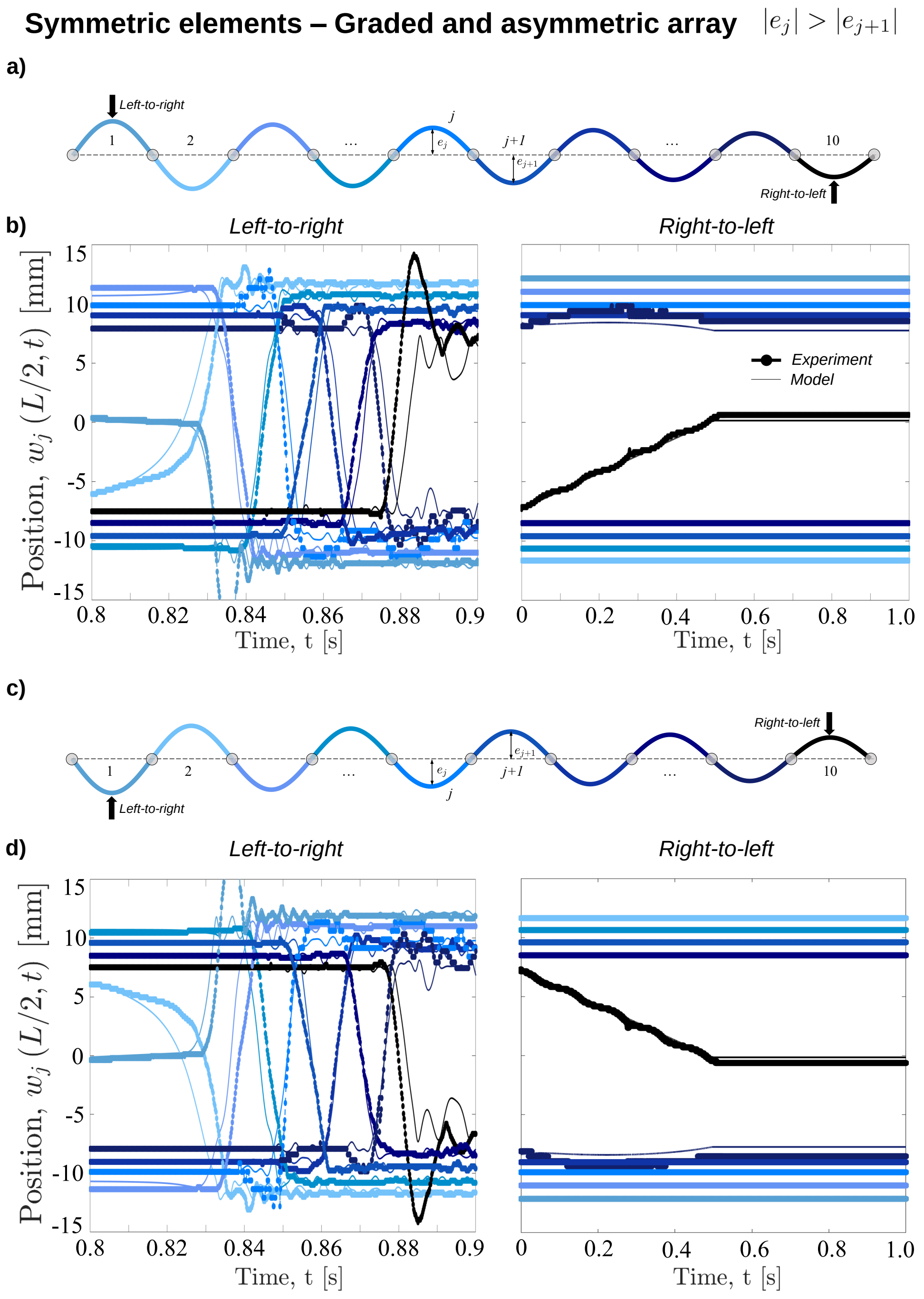

Supplementary Fig. 10: Symmetric elements – Graded and asymmetric array. Results for the same structure considered in Fig. 3 of the main text. **a**, Schematic of the structure. **b**, Comparison between the experimentally measured (thick-dotted lines) and numerically predicted (thin lines) positions of the midpoints of the arches when the system is excited left-to-right and right-to-left as indicated in (a). **c**, Schematic of the structure. **d**, Comparison between the experimentally measured (thick-dotted lines) and numerically predicted (thin lines) positions of the midpoints of the arches when the system is excited left-to-right and right-to-left as indicated in (c).

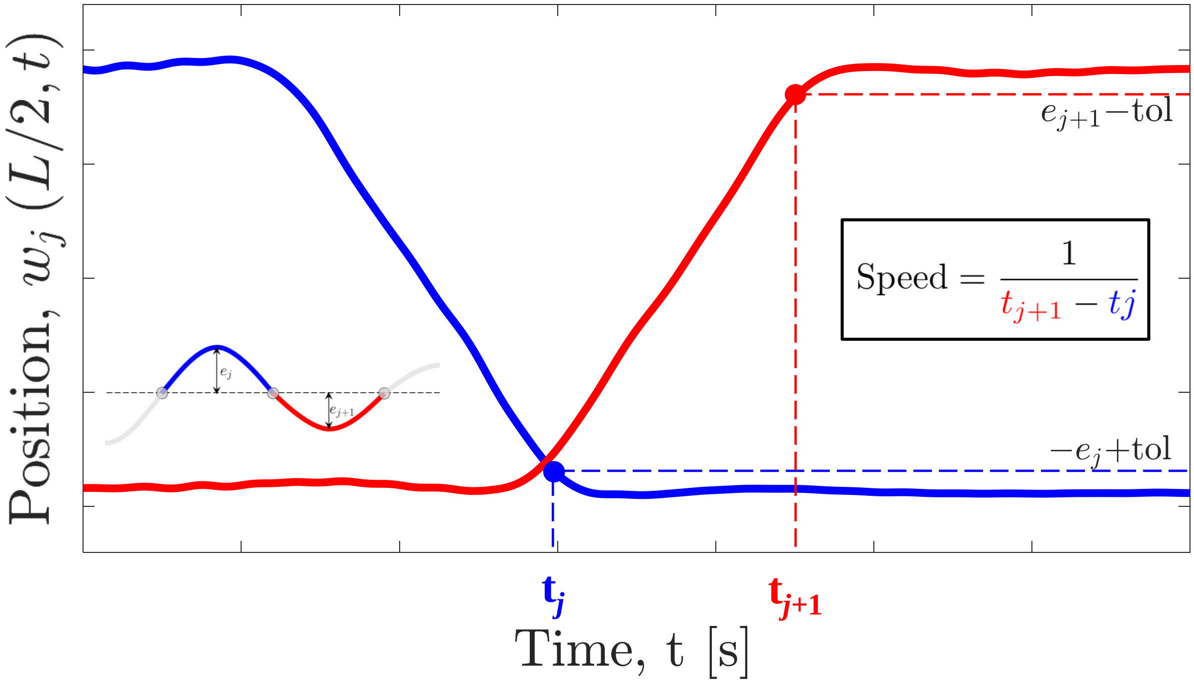

Supplementary Fig. 11: Speed per unit. Schematics illustrating how to compute the local speed in a 1D array of snapping arches.

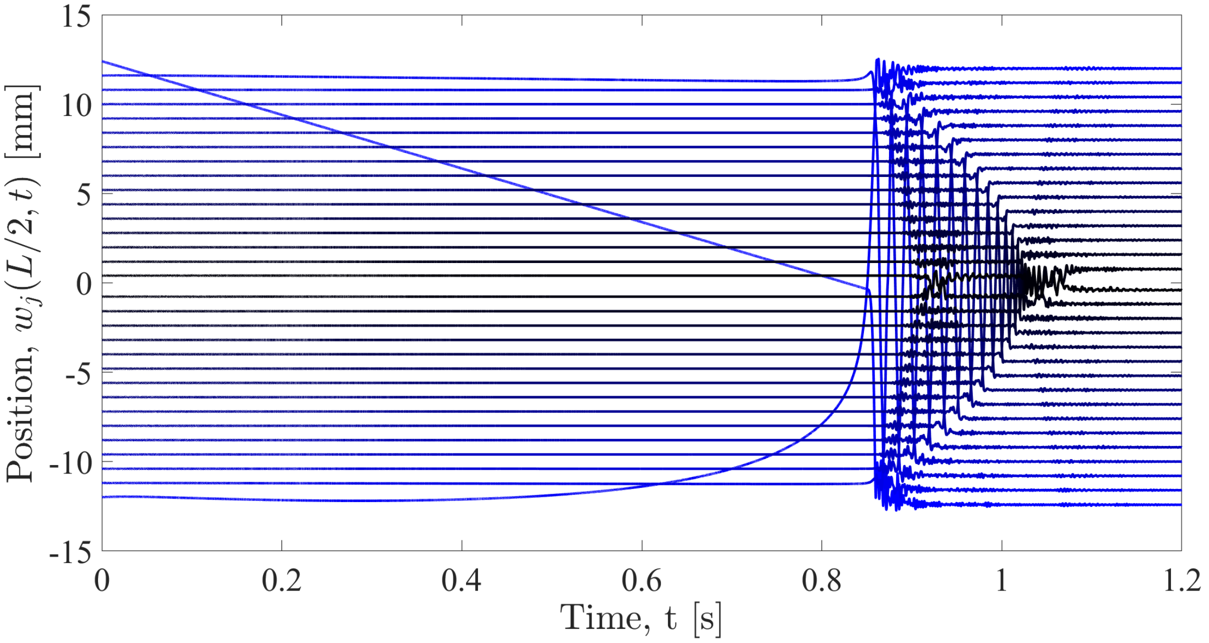

Supplementary Fig. 12: Reversible diode. Numerically predicted positions, $w_j(L/2, t)$, for an array comprising $N = 31$ arches with modulated rise. For this chain $\Delta e_j = 400$m and e_1 = 12.4 mm. The pulse is initiated by pushing down the leftmost arch.

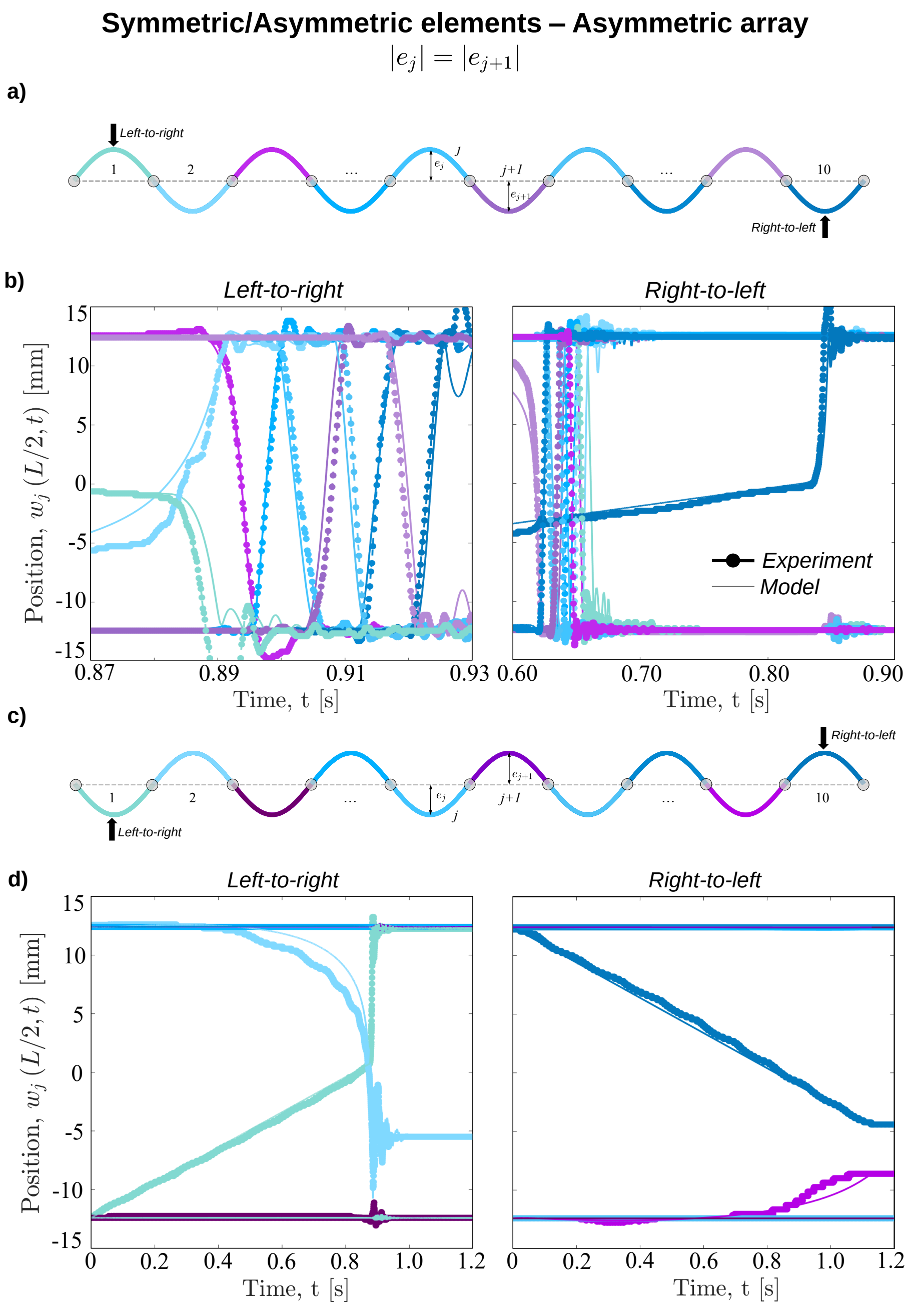

Supplementary Fig. 13: Symmetric/Asymmetric elements – Asymmetric array. Results for the same structure considered in Fig. 4 of the main text. **a**, Schematics of chain. **b**, Comparison between the experimentally measured (thick-dotted lines) and numerically predicted (thin lines) positions of the midpoints of the arches when the system is excited left-to-right and right-to-left as indicated in (a). **c**, Schematics of the chain. **d**, Comparison between the experimentally measured (thick-dotted lines) and numerically predicted (thin lines) positions of the midpoints of the arches when the system is excited left-to-right and right-to-left as indicated in (c). No transition waves are supported as indicated in (d).

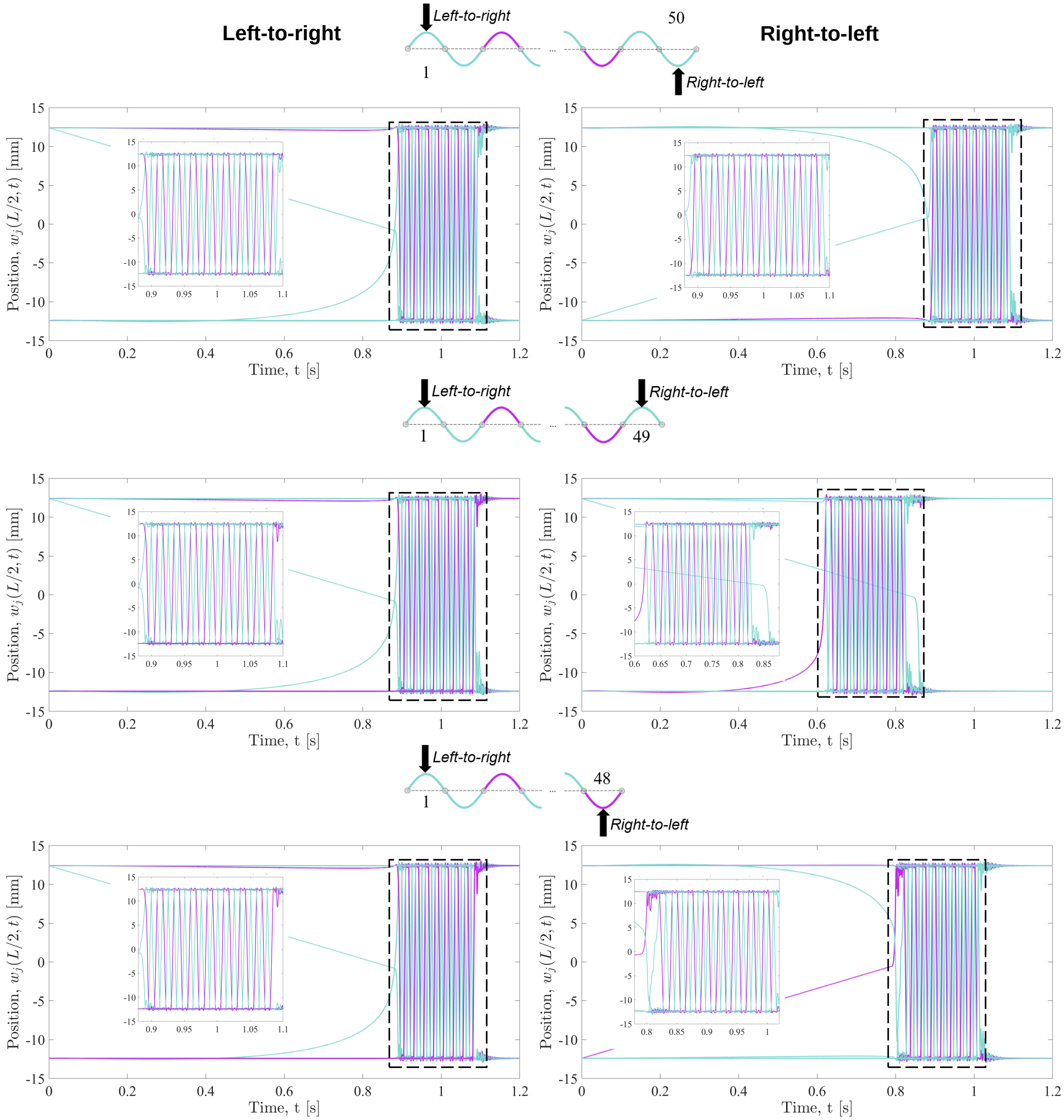

Supplementary Fig. 14: Effect of chain symmetry/asymmetry – Tunable nonreciprocity Numerically predicted positions of the arches' midpoints, $w_j(L/2,t)$, as a function of time for the three chains (comprising $N = 48$, 49 and 50 arches) considered in Fig. 5a of the main text. Results for both left-to-right and right-to-left propagation are shown.

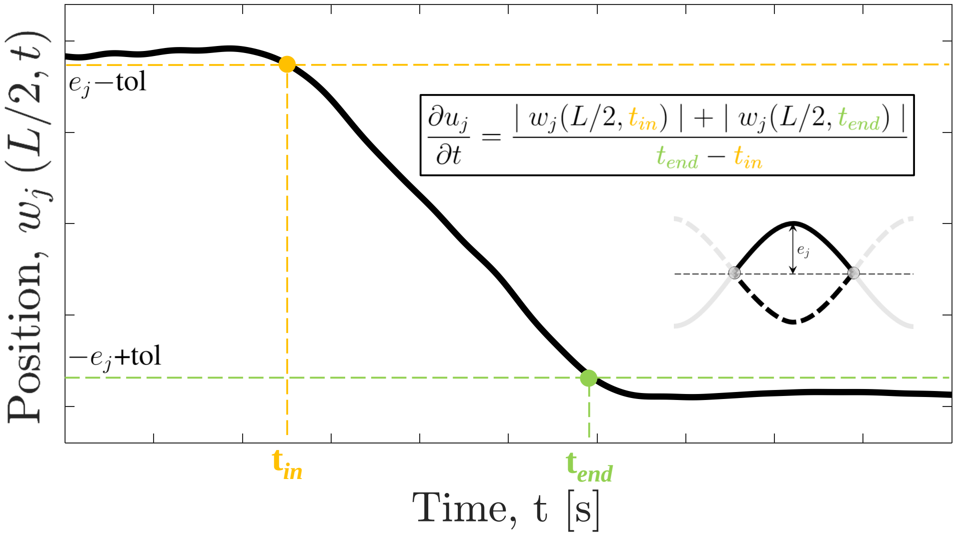

Supplementary Fig. 15: Estimation of c_{global} for a chain comprising only plastically deformed arches. For a chain comprising only elements with asymmetric on-site energy potential the wave speed, c_{global}, can be estimated by balancing the total transported kinetic energy E_d, the difference $\Delta\phi$ between the higher and lower energy well, and the energy dissipated as [13]

$$c_{global} = \frac{2\beta L E_d}{\Delta\phi} \tag{S23}$$

where

$$E_d = \sum_{j=1}^{N} \frac{1}{2} \left(\frac{\partial u_j}{\partial t}\right)^2 L \tag{S24}$$

with $\partial u_j/\partial t$ computed as showed in this figure. Using Eq. (S23) , we find that in a chain comprising $N = 49$ plastically deformed arches with e_j=12.4 mm $c_{global} \sim 503$ units/s while from our simulations we compute a $c_{global} \sim 497$ units/s, with a discrepancy around 1%.

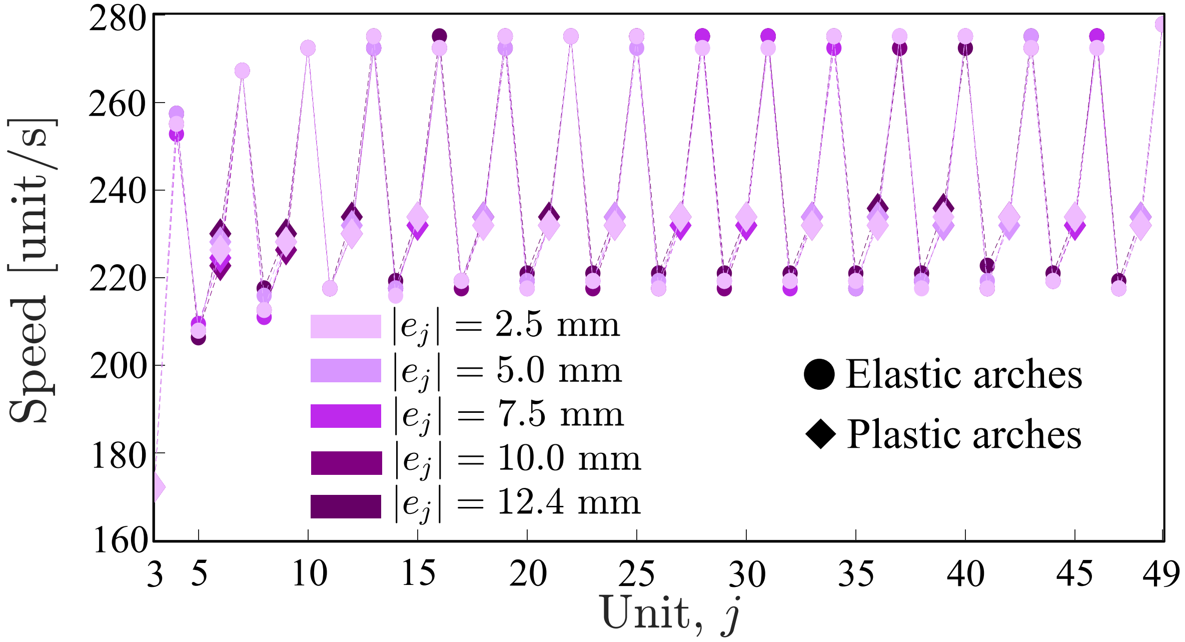

Supplementary Fig. 16: Effect of the arch rise, e_j, on the response of a chain comprising both elastically and plastically deformed arches. Numerically predicted local speed of the transition waves for different rises e_j in a chain comprising $N = 49$ elastically and plastically deformed arches arranged as in Fig. 4b of the main text.

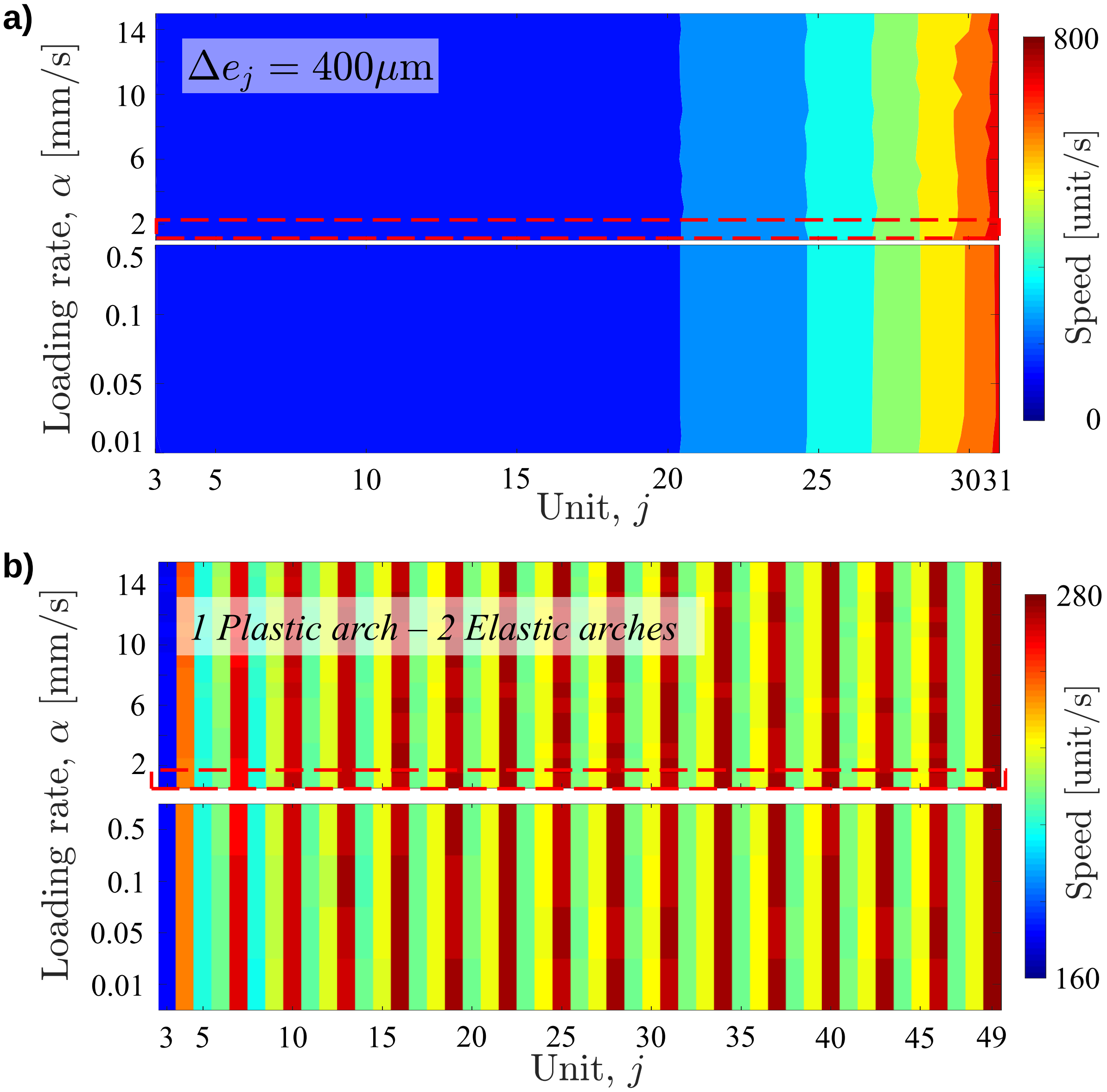

Supplementary Fig. 17: Effect of loading rate α. a, Local speed of the transition waves in a graded system with $\Delta e_j = 400\mu$m and $e_1 = 12.4$ mm for different loading rates α. We find that the wave speed is minimally affected by α. **b**, Local speed of the transition waves for different loading rates α in a system comprising $N = 49$ elastically and plastically deformed arches with $e_j = 12.4$ mm arranged as in Fig. 4b of the main text. Also for this chain we find that the wave speed is minimally affected by α.

.

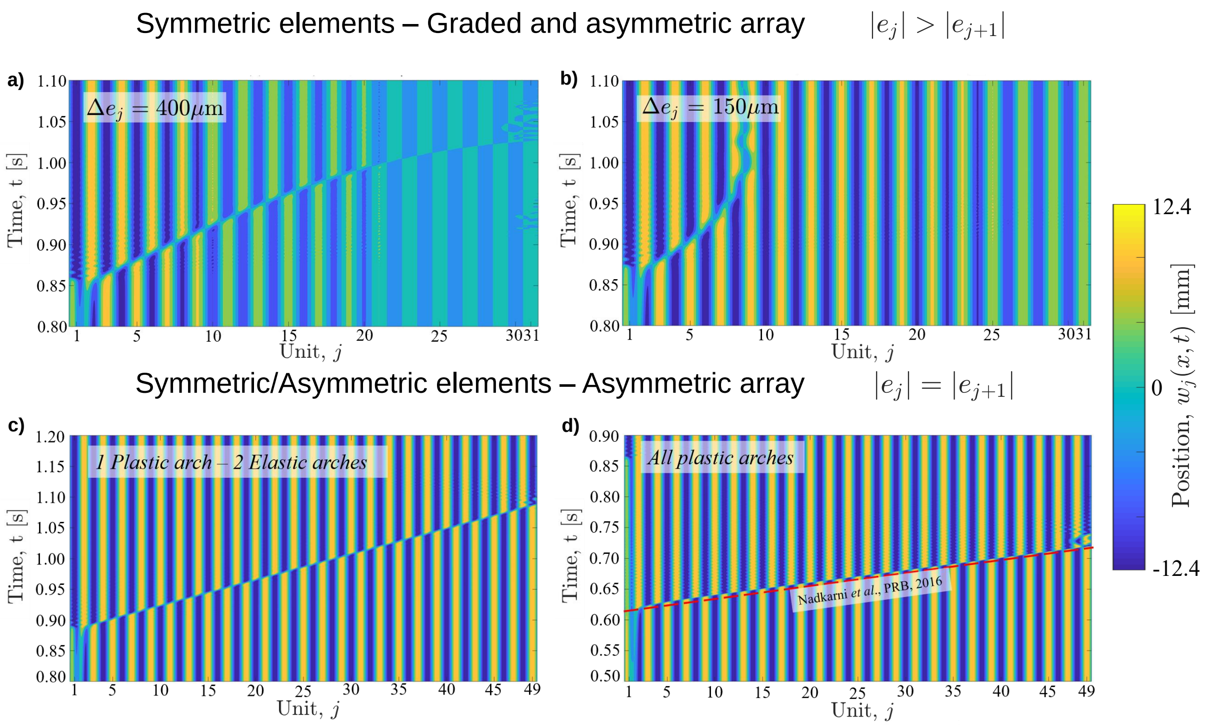

Supplementary Fig. 18: Propagation of transition waves in different chains. Numerically predicted position across each arch, $w_j(x_j, t)$, during the propagation of the wave in (a) a graded chain comprising elastically deformed arches with $\Delta e = 400\mu$m and $e_1 = 12.4$ mm; (b) a graded chain comprising elastically deformed arches with $\Delta e = 150\mu$m and $e_1 = 12.4$ mm; (c) a chain comprising $N = 49$ elastically and plastically deformed arches with $e_j = 12.4$ mm arranged as in Fig. 4b of the main text; (d) a chain comprising $N = 49$ plastically deformed arches with $e_j = 12.4$ mm. The red dashed line in (d) indicate the global speed, c_{global}, predicted by Eq. (S23).

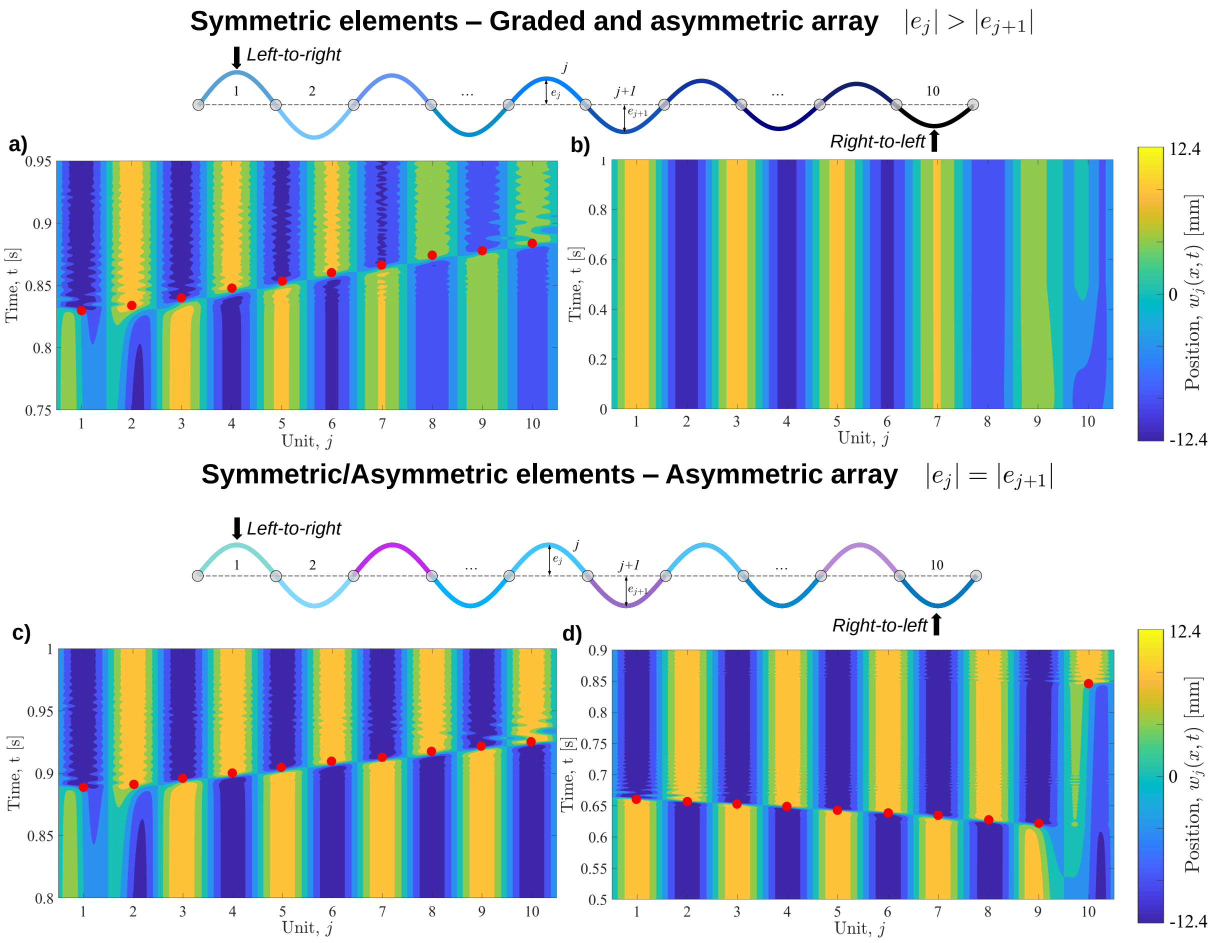

Supplementary Fig. 19: Comparison between experimental and numerical transition waves. Numerically predicted position across each arch, $w_j(x_j, t)$, during the propagation of the wave in (a) the graded chain comprising elastically deformed arches tested in Fig. 3d in the left-to-right direction; (b) same graded chain tested in Fig. 3d in the right-to-left direction; (c) the chain comprising elastically and plastically deformed arches tested in Fig. 4c in the left-to-right direction (pattern showed in the schematic); (d) same chain tested in Fig. 4c in the right-to-left direction. The red dots represent the experimentally measured snapping times for each arch.

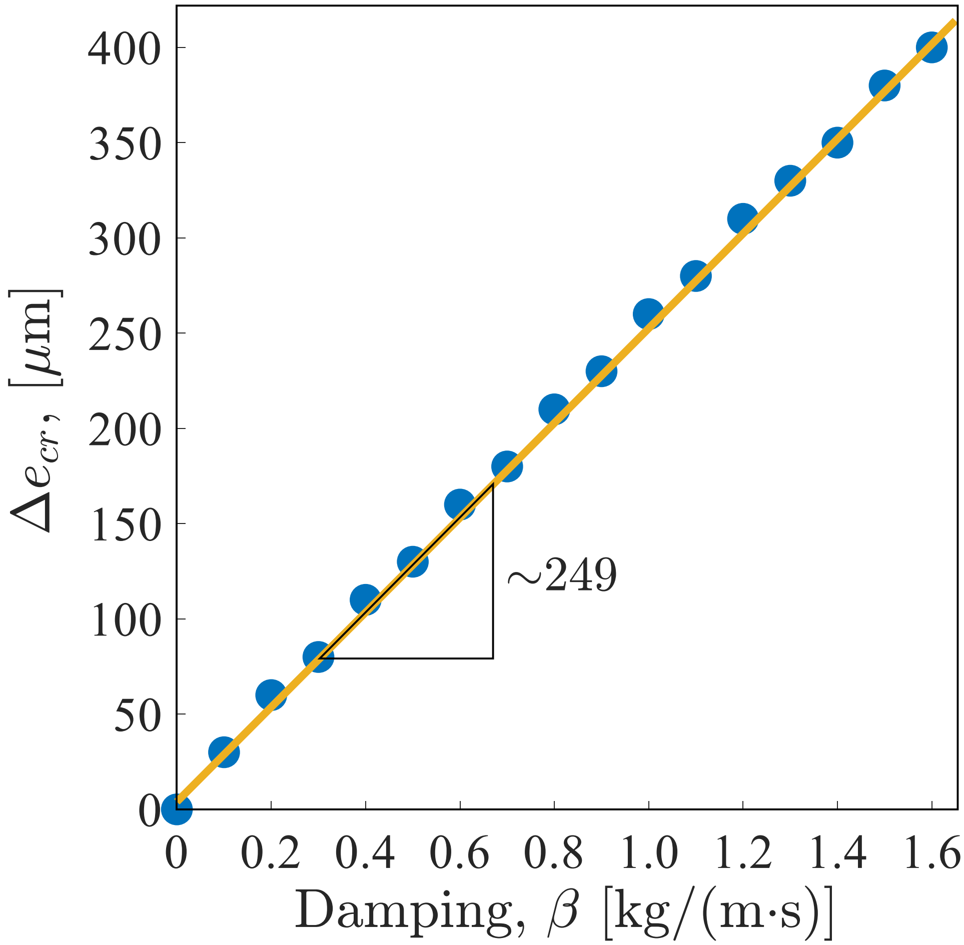

Supplementary Fig. 20: Graded array and damping. Numerically predicted relation between the critical difference in the rises of two consecutive arches, Δe_{cr}, and the viscous damping, β, for a graded and asymmetric array.

S1.7 Supplementary References

[1] Stephen Timoshenko. *History of strength of materials*. Courier Corporation, 1983.

[2] Gabriele Librandi, Eleonora Tubaldi, and Katia Bertoldi. Snapping of hinged arches under displacement control: Strength loss and nonreciprocity. *Phys. Rev. E*, 101:053004, May 2020.

[3] YC Fung and A Kaplan. Buckling of low arches or curved beams of small curvature. *U.S. National Advisory Committee for Aeronautics Technical Note no. 2840, November*, 1952.

[4] Malcom H Lock. Snapping of a shallow sinusoidal arch under a step pressure load. *AIAA Journal*, 4(7):1249–1256, 1966.

[5] AH Nayfeh and DT Mook. *Nonlinear Oscillations*. Wiley, New York, 1979.

[6] Jen-San Chen, Wei-Chia Ro, and Jian-San Lin. Exact static and dynamic critical loads of a sinusoidal arch under a point force at the midpoint. *International Journal of Non-Linear Mechanics*, 44(1):66–70, 2009.

[7] Anupam Pandey, Derek E Moulton, Dominic Vella, and Douglas P Holmes. Dynamics of snapping beams and jumping poppers. *EPL (Europhysics Letters)*, 105(2):24001, 2014.

[8] Michael Gomez, Derek E Moulton, and Dominic Vella. Critical slowing down in purely elastic 'snap-through' instabilities. *Nature Physics*, 13(2):142, 2017.

[9] D.J. Mead. Free wave propagation in periodically supported, infinite beams. *Journal of Sound and Vibration*, 11(2):181 – 197, 1970.

[10] G. Sen Gupta. Natural flexural waves and the normal modes of periodically-supported beams and plates. *Journal of Sound and Vibration*, 13(1):89 – 101, 1970.

[11] Shao-Yu Hung Jen-San Chen. Exact snapping loads of a buckled beam under a midpoint force. *Applied Mathematical Modelling*, 36:1776–1782, 2012.

[12] John S Humphreys. On dynamic snap buckling of shallow arches. *AIAA journal*, 4(5):878–886, 1966.

[13] Neel Nadkarni, Chiara Daraio, Rohan Abeyaratne, and Dennis M. Kochmann. Universal energy transport law for dissipative and diffusive phase transitions. *Phys. Rev. B*, 93:104109, Mar 2016.
