# Supplementary material for: Programming nonreciprocity and reversibility in multistable mechanical metamaterials
Source: Nat Commun. 2021 Jun 8;12:3454. doi: 10.1038/s41467-021-23690-z (PMC8187725; doi:10.1038/s41467-021-23690-z)
Supplement: Supplementary file 3 — Description of Additional Supplementary Files [file 41467_2021_23690_MOESM3_ESM.pdf]

Description of Additional Supplementary Files

File Name: Supplementary Movie 1
Description: Cascade of snapping events in a symmetric array comprising symmetric elements.

File Name: Supplementary Movie 2
Description: Numerical model vs. experimental results for a chain comprising three symmetric elements.

File Name: Supplementary Movie 3
Description: Reversible and nonreciprocal wave propagation in a graded and asymmetric array comprising symmetric elements.

File Name: Supplementary Movie 4
Description: Non-reversible and nonreciprocal wave propagation in an asymmetric array comprising a pattern of symmetric/asymmetric elements.
